# Taking a Bite

**Published:** 1881-09

**Authors:** F. C. Barlow

**Affiliations:** Jersey City


### TAKING A BITE.

*Editor Items*:

Several communications have lately been written regarding the getting of a correct bite or articulation, and its being one of the most important as well as the most difficult objects to obtain. The former I grant, the latter to me is very simple, and the rule *almost infallible*, viz:

After placing the wax for articulation in the mouth, instruct the patient to throw the head slightly up and back, relax the muscles of the jaw and bite. With your thumb and fore finger held lightly upon the chin you can easily discern if the jaw is thrown forward or not; if not *quite certain* have the patient swallow without opening the jaws, if correct, the act of deglutition can be performed without an effort, if wrong he will be obliged to bring the jaw back to its normal position to accomplish the act naturally,

F. C. BARLOW.

JERSEY CITY.

